# Supplementary material for: A Global Screen for Assembly State Changes of the Mitotic Proteome by SEC-SWATH-MS
Source: Cell Syst. 2020 Feb 26;10(2):133–155.e6. doi: 10.1016/j.cels.2020.01.001 (PMC7042714; doi:10.1016/j.cels.2020.01.001)

P27701 | CD82_HUMAN | CD82 KAI1 SAR2 ST6 TSPAN27
Monomer MW [kDa]: 29.626 Monomer expected elution fraction: 51

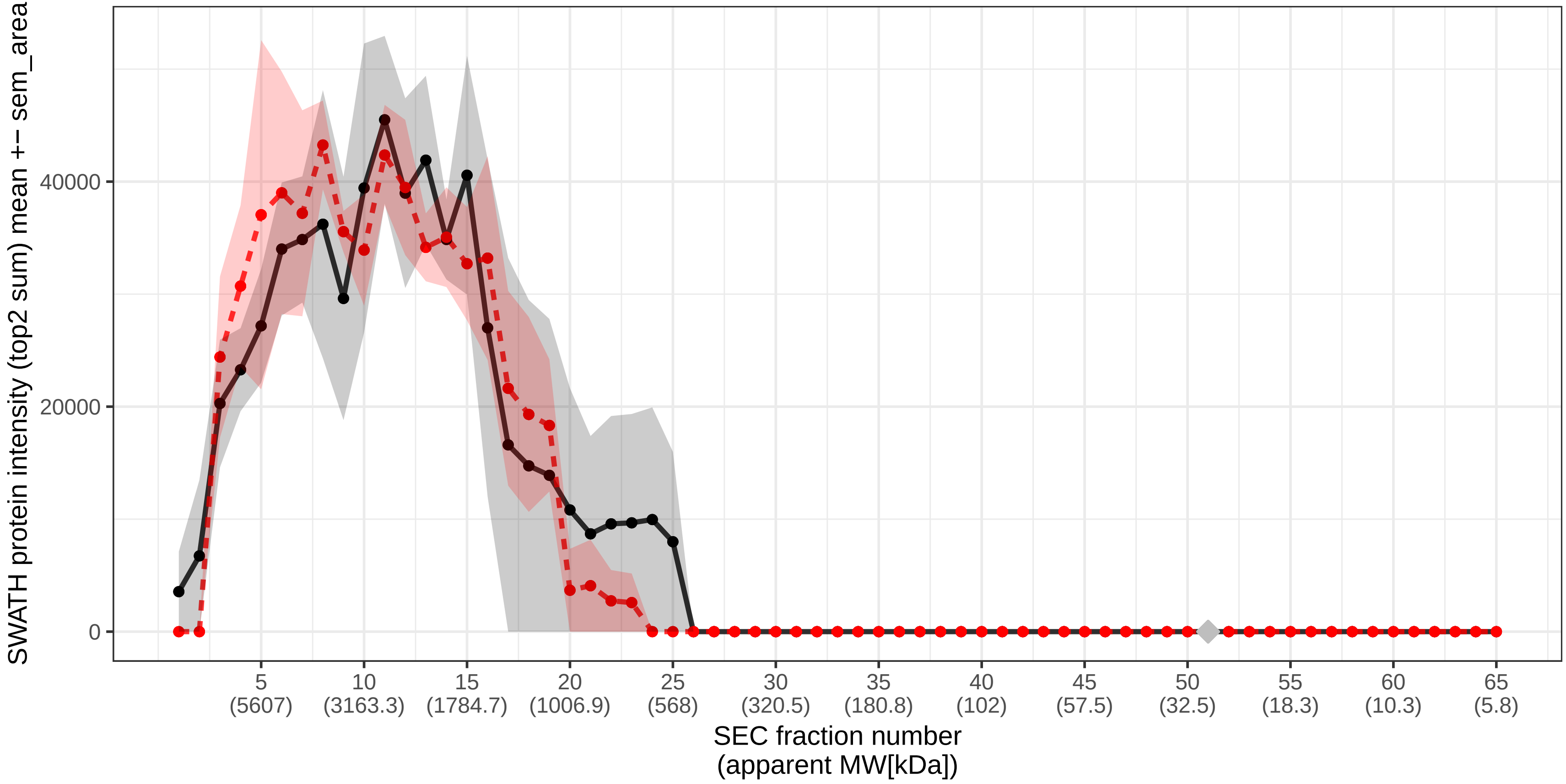

Supplement: Data S1. SEC-SWATH-MS Protein Chromatograms, Related to Figure 1 [file mmc6.zip › SECchrom_P27701_CD82_HUMAN_CD82_KAI1_SAR2_ST6_TSPAN27.pdf]
